# Water-enhanced oxidation of graphite to graphene oxide with controlled species of oxygenated groups†
†Electronic supplementary information (ESI) available. See DOI: 10.1039/c5sc03828f


**DOI:** 10.1039/c5sc03828f

**Published:** 2015-11-26

**Authors:** Ji Chen, Yao Zhang, Miao Zhang, Bowen Yao, Yingru Li, Liang Huang, Chun Li, Gaoquan Shi

**Affiliations:** a Country Collaborative Innovation Center for Nanomaterial Science and Engineering , Department of Chemistry , Tsinghua University , Beijing 100084 , People's Republic of China . Email: gshi@tsinghua.edu.cn ; Fax: +86-10-6277-1149 ; Tel: +86-10-6277-3743

## Abstract

Graphene oxide (GO) sheets with controlled species of oxygen-containing groups are important for fabricating graphene materials with desired structures and properties. Here we report a water-addition modified Hummers method to prepare GO sheets with tunable amounts of hydroxyl and epoxide groups without destroying their structural integrity. This method is simple, effective, and efficient. It can be applied to the mass-production of GO with controlled amounts and species of oxygenated groups, and improve the yields of synthesizing high-quality GO at low temperatures.

## Abstract 2

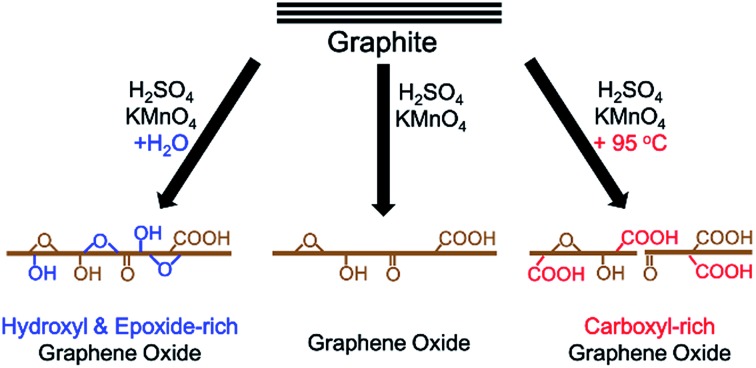
Water-enhanced oxidation of graphite *via* a modified Hummers method can produce graphene oxide with controlled species of oxygenated groups.

## Introduction

Chemically modified graphenes (CMGs), including graphene oxide (GO), reduced graphene oxide (rGO) and their derivatives,^1^ have a variety of applications in electronics,^2^ catalyses,^3^ sensors,^4^ and energy related systems,^5^*etc.*^6^ Among them, GO has been mostly studied, because it is a precursor of other CMGs. A GO sheet has a large amount of oxygenated functional groups (hydroxyl, epoxide, ketone, and carboxyl) on its basal plane and at its edges. Thus, it can be regarded as an amphiphilic macromolecule with huge molar mass. The oxygenated groups of GO provide it with good dispersibility in water, and unique chemical^7^ and supramolecular^8^ properties. They are also active sites for the covalent functionalization of GO with small organic molecules, polymers, or inorganic nanoparticles to realize various applications.^9^ In fact, the selective formation of functional groups on GO sheets is important for realizing its effective and specific functionalization: (1) specific oxygenated groups on GO can satisfy the requirements of grafting functional species with matching functional groups. For example, carboxyl groups can be converted into esters^10^ or amides^11^ by reacting with the molecules containing hydroxyl or amino groups. Covalent C–N bonds can be formed by opening epoxy groups with amines;^12^ vicinal hydroxyl groups can be utilized to modify GO with molecules containing boronic acid groups *via* forming boronic esters;^13^ (2) specific functional groups determine the grafting sites on GO sheets: carboxyl or hydroxyl/epoxide groups enable the functionalization of GO sheets mainly at their edges or on their basal planes;^14^ (3) carboxyl groups can be preserved after reduction, providing negative charges *via* ionization to improve the dispersibility of rGO in aqueous media.^15^ The oxygenated groups of GO can be partially removed by reduction to restore its conjugated structure. However, the holes and edges of GO sheets are unable to be restored to a graphitic structure, strongly decreasing their mechanical, thermal, and electrical properties.^16^ Therefore, a cheap, convenient, and effective technique for the mass-production of GO with less permanent defects and controlled species of oxygenated groups is important for achieving high-quality CMGs.

GO can be prepared by oxidation and exfoliation of graphite. The Hummers method is the most widely employed technique for this purpose.^17^ In this method, H_2_SO_4_ and NaNO_3_ act as intercalation reagents of graphite, and KMnO_4_ oxidizes the acid-intercalated graphite into graphite oxide (GrO). However, the use of NaNO_3_ leads to the formation of NO_2_/N_2_O_4_ toxic gases, and introduces Na^+^ and NO_3_^–^ ions to the waste water. Recently, Tour *et al.* improved the Hummers method by excluding NaNO_3_, increasing the amount of KMnO_4_, and performing the reaction in a 9 : 1 H_2_SO_4_/H_3_PO_4_ mixture for a prolonged time.^18^ This method avoids releasing toxic gases, and can be used to produce heavily oxidized hydrophilic GO in high yield. Unfortunately, the structural integrity of the GO sheets was severely destroyed as indicated by its high content of carboxyl groups. More recently, our group revealed that the removal of NaNO_3_ from the chemical recipe of the Hummers method did not affect the yield and oxidation degree of GO.^19^ This modified method partly addressed the environmental issues of the Hummers method. Nevertheless, none of the techniques described above can be used to control the relative contents of functional groups on GO sheets.

On the other hand, GO with a high degree of oxidation usually has a high content of permanent defects.^18^,^20^ Fortunately, Eigler and coworkers recently reported that maintaining a low reaction temperature (<5–10 °C) during both the oxidation of graphite and the post-treatment of GO could reduce the possibility of forming impossible-to-heal holes in GO sheets. This method can synthesize GO with greatly improved quality.^21^ However, its procedures are complicated and time-consuming, and the yield of GO is low. Nevertheless, this excellent work provided an effective approach for the chemical synthesis of high-quality GO.

**Scheme 1:**
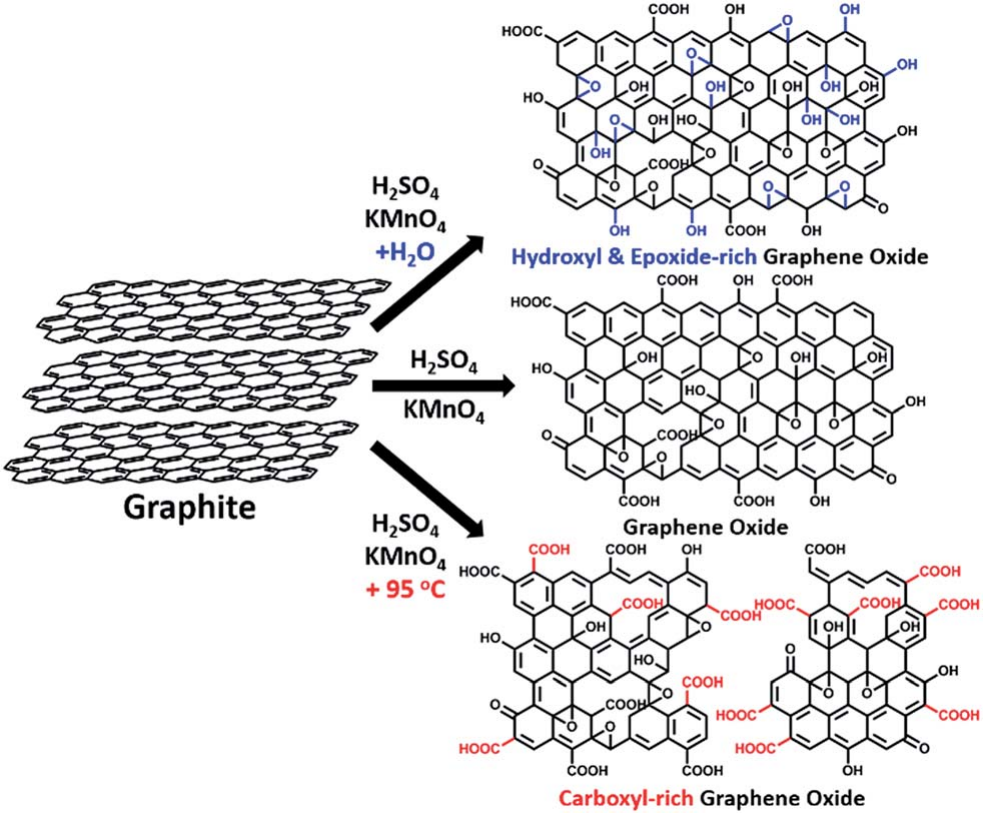
The synthesis of GOs with controlled species of oxygenated groups.

In this paper, we report that heavily oxidized GO with good structural integrity can be produced in a high yield by adding a certain amount of water to the reaction system of our modified Hummers method.^19^ Furthermore, the content of hydroxyl/epoxide or carboxyl groups on the GO sheets can be modulated by controlling the content of water in the reaction system or the destructive oxidizing of GrO at 95 °C in the presence of a large amount of water and the remaining Mn(vii) compound (Scheme 1). This method can also be applied to significantly increase the yield of high-quality GO prepared at low temperatures. This ‘water-enhanced oxidation’ is attributed to the formation of strong oxidative radicals (hydroxyl radical or atomic oxygen) by the Mn-catalyzed decomposition of O_3_ that is generated by the oxidation of water with Mn(vii) compound in the H_2_SO_4_ solution of KMnO_4_.

## Results and discussion

GO samples were prepared *via* a modified Hummers method^19^ with the initial addition of different volumes of water to the reaction systems. Typically, 1.0 g graphite powder was oxidized by KMnO_4_ (3.0 g) in 46 mL concentrated H_2_SO_4_ containing *n* mL water at 40 °C for 2 h, and the resulting GO is nominated as GO-*n* and the corresponding reduced GO is named rGO-*n*. A control GO sample, GO-0-95, was synthesized in the system without the initial addition of water. However, after the oxidation process, 100 mL water was slowly added into the reaction system, and kept at 95 °C for 15 min. The corresponding reduced GO is called rGO-0-95.

All of these GO samples were carefully purified for characterization (see Methods in ESI†).

**Fig. 1:**
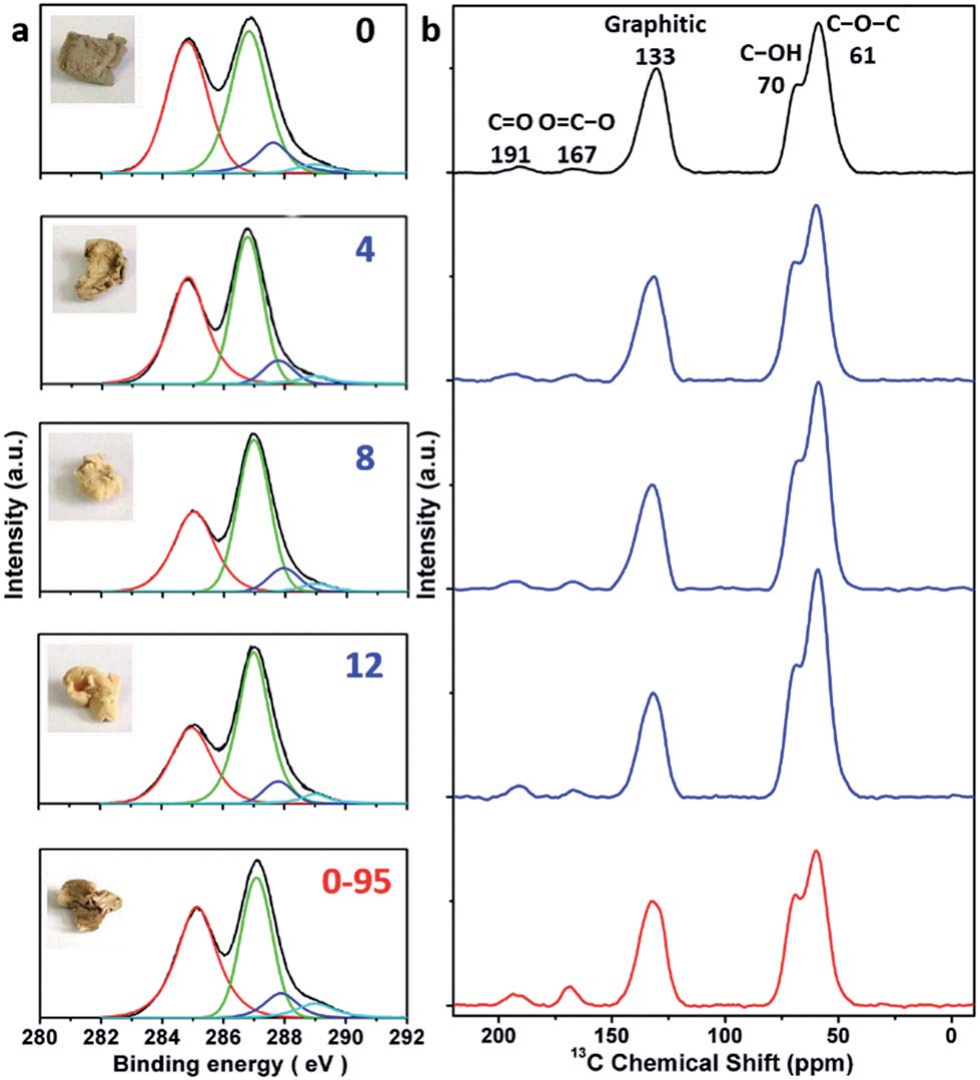
(a) C 1s XPS spectra and photographs (inset), and (b) magic-angle spinning ^13^C ssNMR spectra of different freeze-dried GO-*n* and GO-0-95 samples; the values of *n* are depicted in (a).

The delocalized π-conjugated structure of a graphene sheet is gradually fragmented to smaller domains upon functionalization, weakening its absorption of visible light. Thus, the color difference between GO samples can be used to qualitatively compare their functionalization degree.^17^ Experimentally, the ‘water-enhanced oxidation’ of graphite to GO can be directly indicated by the different colors of the freeze-dried GO samples (insets of Fig. 1a). They became brighter in the sequence of GO-0 to GO-12, reflecting the increase in their oxidative functionalization. This conclusion was further supported by structural analysis. The X-ray photoelectron spectroscopy (XPS) C 1s spectrum of each GO sample consists of four types of carbon bonds: C–C/C=C (284.6 eV), C–O (286.6 eV), C=O (287.8 eV), and O–C=O (289.0 eV).^19^ The peak intensity ratio (*I*_OC_/*I*_CC_) of oxygenated carbon atoms (C–O, C=O, and O–C=O) and intact carbon (C–C and C=C) reflects the oxidation degree of GO,^18^ and this value increases in the following sequence: GO-0 (1.15) < GO-4 (1.27) < GO-8 (1.69) < GO-12 (2.02) (Fig. 1a). The XPS C 1s analysis of the GO samples synthesized by the stepwise increase of the volume of water by 2 mL showed the same results (Fig. S1†), but the increment of *I*_OC_/*I*_CC_ becomes less pronounced as the water volume > 10 mL. The ‘water-enhanced oxidation’ was also confirmed by the magic-angle spinning ^13^C solid-state nuclear magnetic resonance (ssNMR) spectra of the as-prepared GO samples. The ^13^C ssNMR spectrum of GO mainly has the following signals: epoxide (C–O–C, ∼61 ppm), hydroxyl (C–OH, ∼70 ppm), graphitic sp^2^ carbon (C=C, ∼133 ppm), carboxylic acid carbonyl (O–C=O, ∼167 ppm), and ketone carbonyl (C=O, ∼191 ppm).^20^,^22^–^24^ All the spectra shown in Fig. 1b were normalized with respect to the intensity of the signal of graphitic sp^2^ carbon at 133 ppm (Fig. 1b). Among the oxygenated groups, O–C=O and C=O are mainly located at the edges of basal-plane vacancies or at the periphery of the GO sheets;^14^,^24^ thus their contents reflect the relative amounts of permanent defects. The fewer the permanent defects, the better the structural integrity of the GO sheets. The spectrum of GO-0 is similar to those of the GO samples reported in literature^20^,^22^–^24^ except for its relatively weaker peaks of the O–C=O and C=O groups, reflecting a better structural integrity. According to Fig. 1b, the epoxide and hydroxyl signals increase significantly in the sequence of GO-0 < GO-4 < GO-8 < GO-12, while the other signals have similar intensities. This result indicates that the addition of water during oxidation led to the selective formation of epoxide/hydroxyl groups without damaging the structural integrity of the GO sheets. In a sharp contrast, the XPS C 1s spectrum of GO-0-95 shows a higher oxidation degree compared with that of GO-0. This difference is mainly attributed to the relatively high content of carboxyl groups in GO-0-95 as indicated by the significantly enhanced and downfield shifted signal of O–C=O (∼169 ppm) in its ssNMR spectrum.^23^ On the basis of the above observations, it is reasonable to conclude that the initial addition of water selectively increased the content of hydroxyl and epoxide groups of GO, while the additional 95 °C process with a large amount of water mainly increased the content of carboxyl groups, which is destructive to the graphitic structure.

**Fig. 2:**
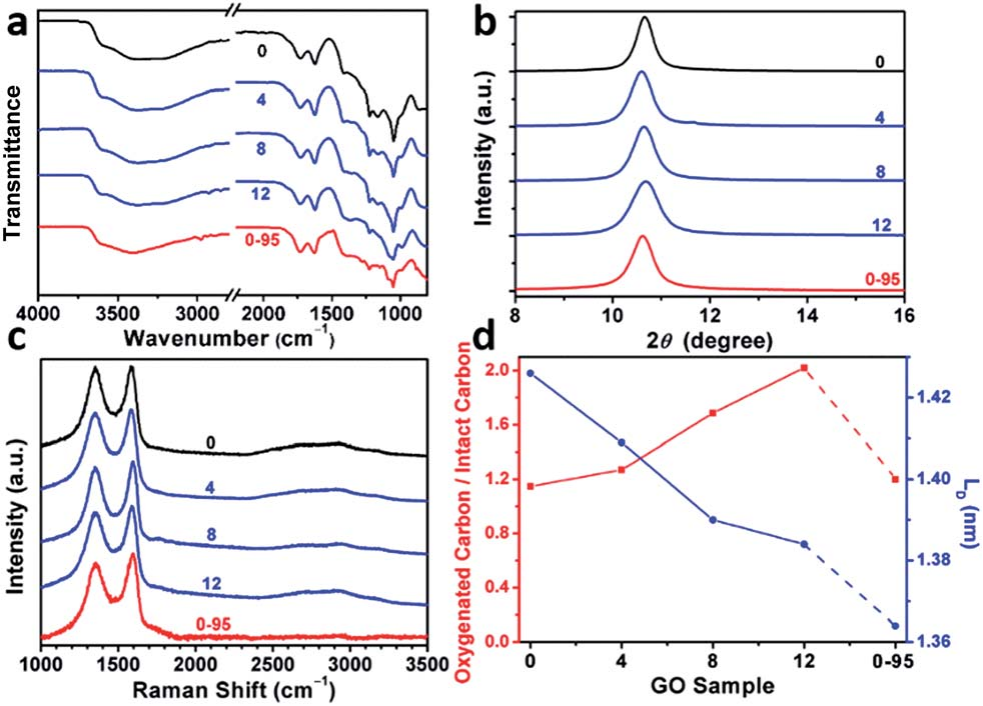
(a) ATR-FTIR spectra, (b) XRD patterns, (c) 514.5 nm excited Raman spectra, (d) atomic ratio of oxygenated carbons/intact carbons and mean distance between two defects (*L*_D_) of GO-*n* and GO-0-95; the values of *n* are depicted in the figure.

The modulation of the oxidation degree and functional groups of GO by adding water has also been confirmed by ATR-FTIR spectral studies (Fig. 2a). The intensity ratio of the C=O (1740–1720 cm^–1^)/H_2_O (∼1620 cm^–1^) peaks remains nearly unchanged in the spectra of GO-0 to GO-12, while this ratio for the spectrum of GO-0-95 is much higher, indicating the last sample has the highest content of carbonyl groups. However, the other oxygenated groups (C–O–C, ∼1000 cm^–1^; C–O, 1230 cm^–1^; O–H, 3600–3300 cm^–1^) exhibited comparable intensities in all of the IR spectra.^18^,^25^ The X-ray diffraction patterns of the GO samples (Fig. 2b and S2†) show peaks centered at about 2*θ* = 10.65°, and their *d*-spacings were calculated to be around 8.30 Å. This value is much larger than that of natural graphite (3.35 Å), indicating the successful functionalization of graphene by oxygenated groups.^26^ Moreover, the full widths at half maximum (FWHM) of the XRD peaks widened upon increasing the volume of water from 0 to 14 mL: GO-0 (0.42°), GO-4 (0.52°), GO-8 (0.55°), and GO-12 (0.66°), followed by a decrease for GO-0-95 (0.55°). The FWHM has a positive correlation with the oxidation degree of GO.^14^

Raman spectroscopy is a powerful tool for studying the structures of CMGs. The typical Raman spectrum of CMG sheets consists of the D-, G-, and 2D-bands of carbon. The D-band (1330–1340 cm^–1^) is associated with the defect-activated breathing modes of six-membered carbon rings, and the G-band (1580–1600 cm^–1^) is assigned to the E_2g_ phonons at the Brillouin zone center.^27^,^28^ Specifically, the intensity ratio of D- to G-bands, *I*_D_/*I*_G_, reflects the average distance between defects (*L*_D_) in graphene. For graphene and its derivatives, the value of *I*_D_/*I*_G_ initially increases with increasing *L*_D_ (1–3 nm, stage 2), followed by a decrease (>3 nm, stage 1).^29^ Usually, GO and rGO belong to stage 2. The defects in GO can be divided into two types as illustrated in Fig. S3:† (1) defects induced by removable oxygenated groups that can be partly restored to graphitic structure upon reduction; and (2) permanent vacancies that are impossible-to-heal by reduction.^29^ The Raman spectrum of GO can indicate its overall defect density by calculating the *L*_D_ derived from *I*_D_/*I*_G_. However, the relative amounts of these two types of defects in GO have to be evaluated by combining the ssNMR and XPS spectra of GO (indicative of the content of functional groups) and the Raman spectrum of the corresponding rGO (indicative of the permanent defects).

In Fig. 2c and Fig. S4,† the Raman spectra of all the samples feature broad and merged D- and G-bands, typical for GO. The *I*_D_/*I*_G_s of GO-0, GO-4, GO-8, GO-12, and GO-0-95 were measured to be 0.997, 0.963, 0.931, 0.920, and 0.886, respectively. The trend of decreasing *I*_D_/*I*_G_s indicates the decrease of *L*_D_s in GO sheets. Actually, the *L*_D_s of GO-0 to GO-12 were calculated to be 1.43, 1.41, 1.39, and 1.38 nm, correspondingly (Fig. 2d). Considering the increasing degree of oxidation as described above, the Raman results indicate that the functionalization-induced defects of GO sheets increase with the volume of initial added water. This conclusion has also been confirmed by the increase of *I*_OC_/*I*_CC_ from 1.15 for GO-0 to 2.02 for GO-12. Surprisingly, GO-0-95 has a small *I*_OC_/*I*_CC_ (1.20), while its *L*_D_ (1.26 nm) is the smallest among these GO samples. This result indicates that the ‘defects’ of GO-0-95 are mainly originated from permanent vacancies, implying that the reaction at 95 °C severely destroyed the graphitic domains of the GO sheets. This conclusion was also supported by its relatively higher content of carboxyl groups that usually locate at the permanent defects (vacancies and edges).^14^,^30^

**Fig. 3:**
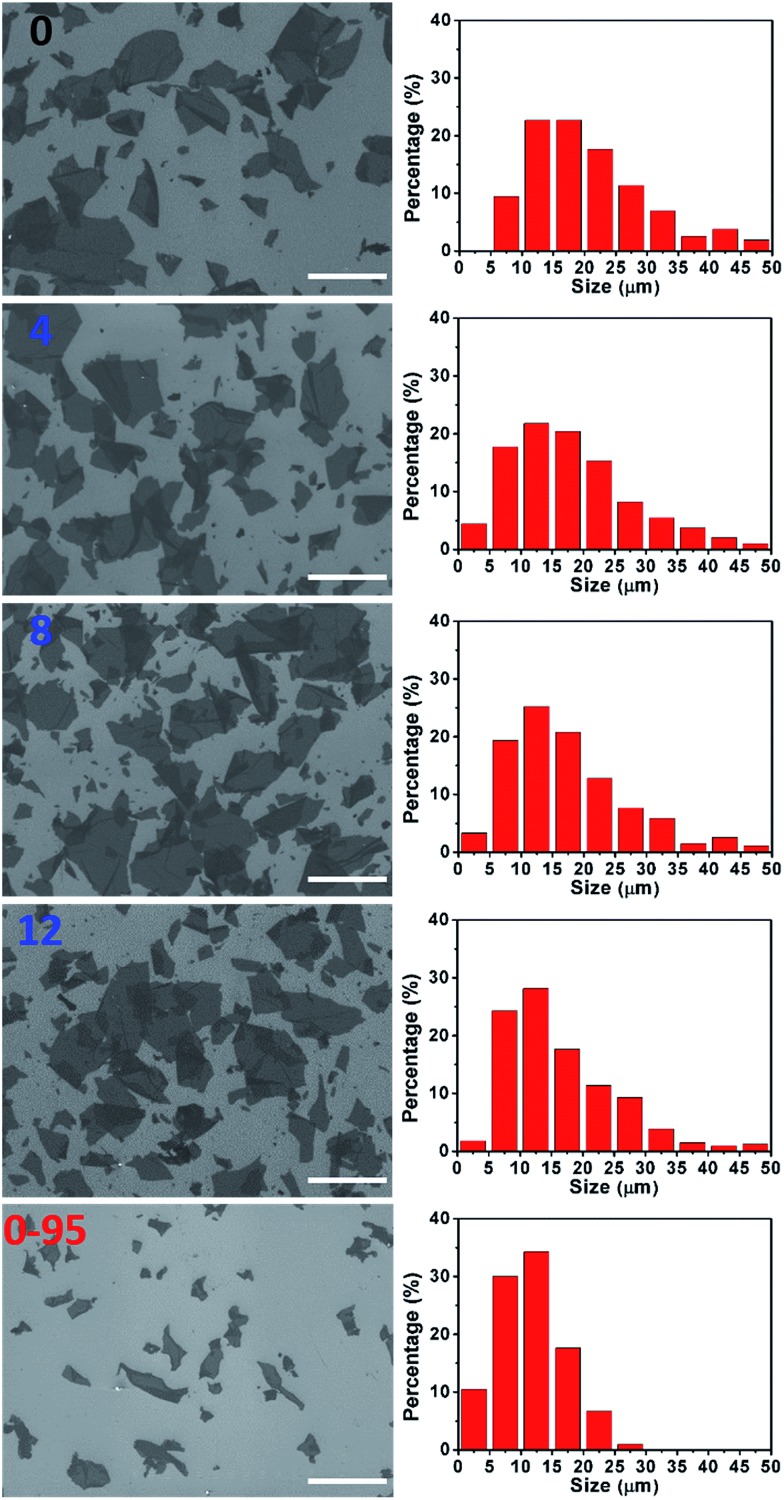
Typical SEM images and corresponding histograms of GO size distributions (right to the SEM image) of GO-*n* and GO-0-95 samples; the values of *n* are depicted in the panels; scale bar, 50 μm. The histograms of GO size distributions were obtained by counting more than 200 sheets for each sample.

The severe structural damage of the GO-0-95 sheets was also indicated by the sheer decrease in their lateral dimensions. The sizes of the GO-0 to GO-12 sheets have a wide distribution from <5 up to over 50 μm, mainly (>90%) in the range 5–40 μm (Fig. 3). The average sizes of GO-*n* (*n* = 0, 4, 8, 12) were measured to be 19.6, 18.0, 16.5, and 16.2 μm, respectively. The gradual decrease of size was caused by the unavoidable cutting of GO sheets upon water-enhanced oxidation.^31^ In GO-0-95, however, no sheet was found to be larger than 30 μm, and most of them (>90%) are in the range 0–25 μm. The average size of the GO-0-95 sheets was measured to be 11.8 μm, only about 60% that of GO-0. This result indicates that the GO sheets experienced severe cutting by oxidation with the remaining Mn(vii) species and/or decomposition at an elevated temperature of 95 °C.^25^ The fragmentation of the GO-0-95 sheets is consistent with the structural characterization results discussed above.

**Fig. 4:**
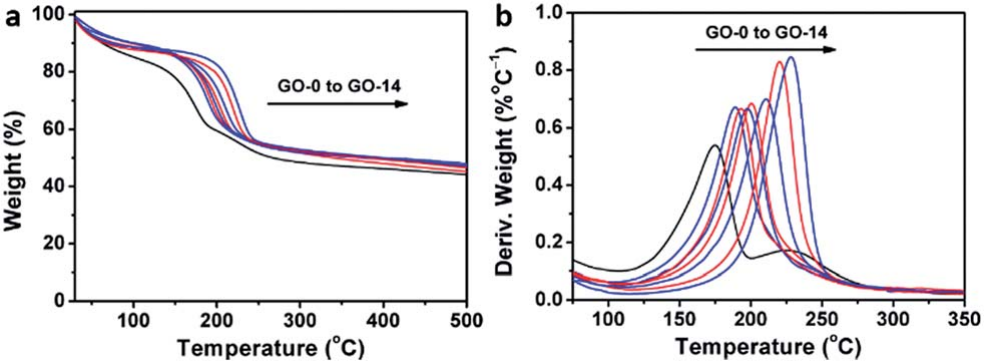
(a) Thermogravimetric analysis curves of different GO samples, and (b) the corresponding derivative thermogravimetric curves.

Interestingly, thermogravimetric analysis (TGA, Fig. 4a) demonstrated that the decomposition temperature (*T*_d_) of a GO sample increased with its oxidation degree. This trend agrees well with that of partially reduced GO samples.^32^ As shown in Fig. 4b, the peak temperature of weight loss increases gradually from 174.6 °C for GO-0 to 228.0 °C for GO-14. In fact, the decomposition of GO is a disproportionation reaction, producing rGO and gaseous CO, CO_2_, and H_2_O.^25^ This gas-formation process requires overcoming the strong interlayer hydrogen bonding, making the *T*_d_ of GO increase with its oxidation degree. The *T*_d_ of GO-0-95 was measured to be 208.5 °C (Fig. S5†), between those of GO-8 (204.0 °C) and GO-10 (210.6 °C). Considering the comparable oxidation degrees of GO-0-95 and GO-4, the relatively higher *T*_d_ of the former can be explained by the stronger hydrogen bonding ability of its carboxyl groups. It should be noted here, the derivative thermogravimetric curve of GO-0 has double peaks, and the additional peak is attributed to its residual organosulfate.^33^ The peak related to organosulfate is negligible in the curve of GO-*n* (*n* = 2 to 14) or GO-0-95, indicating that water in the reaction system significantly promoted the hydrolysis of organosulfate.

**Fig. 5:**
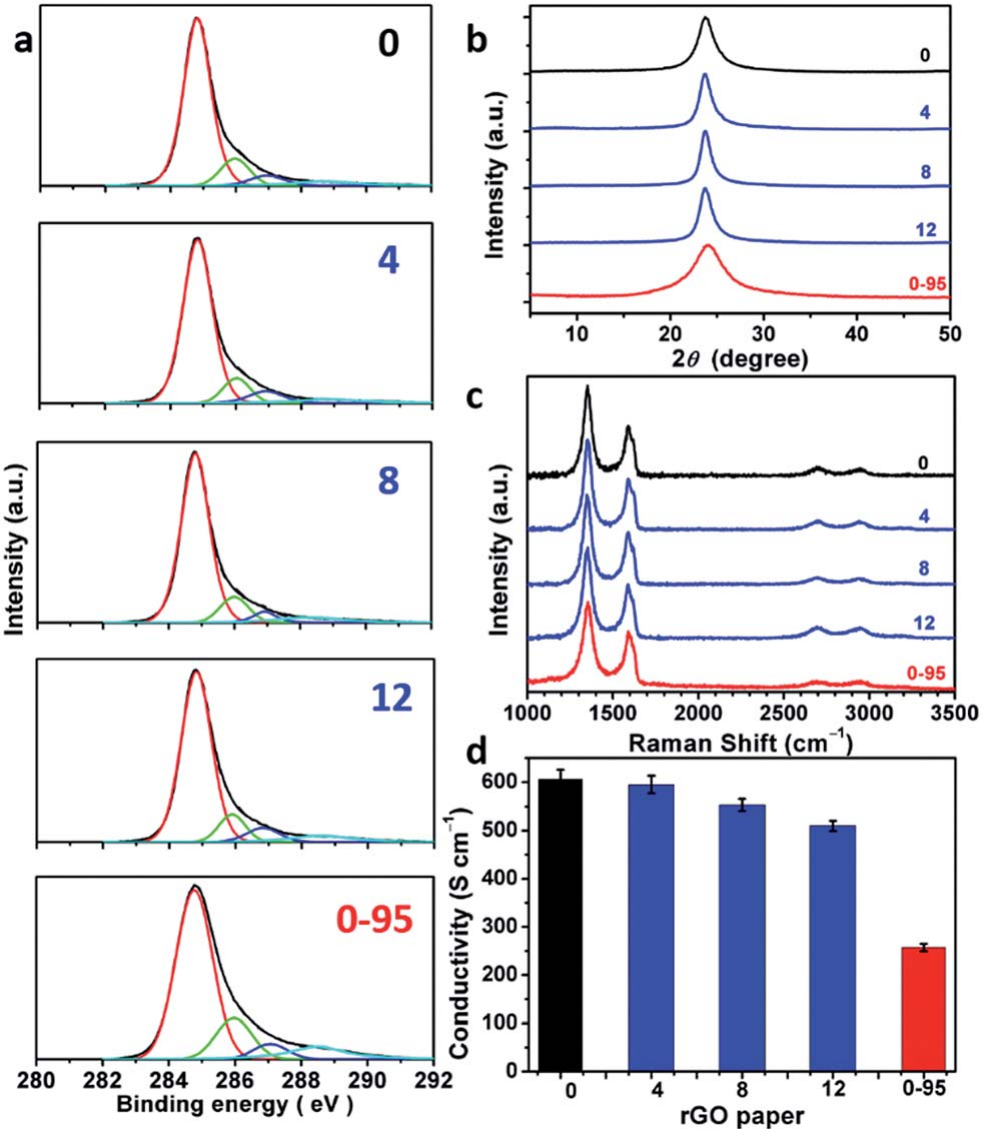
(a) C 1s XPS spectra, (b) XRD patterns, (c) 514.5 nm excited Raman spectra, (d) conductivities of rGO-*n* and rGO-0-95 papers; the values of *n* are depicted in the figure.

rGO papers were prepared by reducing GO papers with HI dissolved in a water/ethanol mixed solvent (v/v = 1 : 1). XPS C 1s analysis indicates that most of the oxygenated groups of GO have been removed upon reduction (Fig. 5a and Fig. S6†). The spectra of rGO-0 to rGO-12 have similar features, while the spectrum of rGO-14 shows a relatively stronger peak of carboxyl groups caused by a slight excess oxidation. In sharp contrast, rGO-0-95 exhibited significantly higher content of carboxyl groups at permanent vacancies.^14^ The XRD patterns (Fig. 5b and S7†) of all the rGO papers exhibit a single characteristic (002) reflection peak at similar positions (2*θ* = 23.8–24.0°, *d*-spaces = 3.71–3.74 Å). However, the XRD peak of rGO-0-95 is much broader (FWHM = 4.0°) than those of rGO-*n* (FWHM = 1.7–2.3°), also reflecting its higher content of residual oxygenated groups. The typical Raman spectra of all the rGO papers (Fig. 5c and S8†) feature stronger D-bands with respect to G-bands, indicating the partial restoration of graphitic structures.^29^ The *I*_D_/*I*_G_s of rGO-0 to rGO-12 slightly decreased from 1.79 to 1.77, 1.74, or 1.68, while that of rGO-0-95 sharply decreased to only 1.52. The corresponding *L*_D_s were calculated to be 1.88, 1.87, 1.85, 1.82, and 1.73 nm. These data further confirm that the decreasing of the corresponding *L*_D_s from GO-0 to GO-12 is mainly attributed to the increase of their epoxy and hydroxyl groups, while the low *L*_D_ of GO-0-95 is mainly a result of its impossible-to-heal holes or vacancies with carboxyl capping groups. However, it should be noted that too much water addition (≥6 mL) led to forming relatively more permanent defects to decrease the *L*_D_ of rGO. The structural difference between the rGO papers is also reflected by their conductivities (Fig. 5d and S9†). The conductivity of the rGO papers slightly decreased in the sequence of rGO-0 (605 ± 20 S cm^–1^) > rGO-4 (595 ± 18 S cm^–1^) > rGO-8 (554 ± 13 S cm^–1^) > rGO-12 (510 ± 11 S cm^–1^). Accordingly, a small amount of water addition (≤4 mL) during oxidation has negligible effect on the structural integrity of GO and the corresponding rGO, while a large amount of water (≥6 mL) led to a slight decrease of their quality because of excess oxidation. Notably, rGO-0-95 exhibited a much lower conductivity (257 ± 8 S cm^–1^), only about 40% of that of the rGO-0 papers. This is mainly due to the smaller sizes and much higher content of the permanent defects of rGO-0-95.

**Fig. 6:**
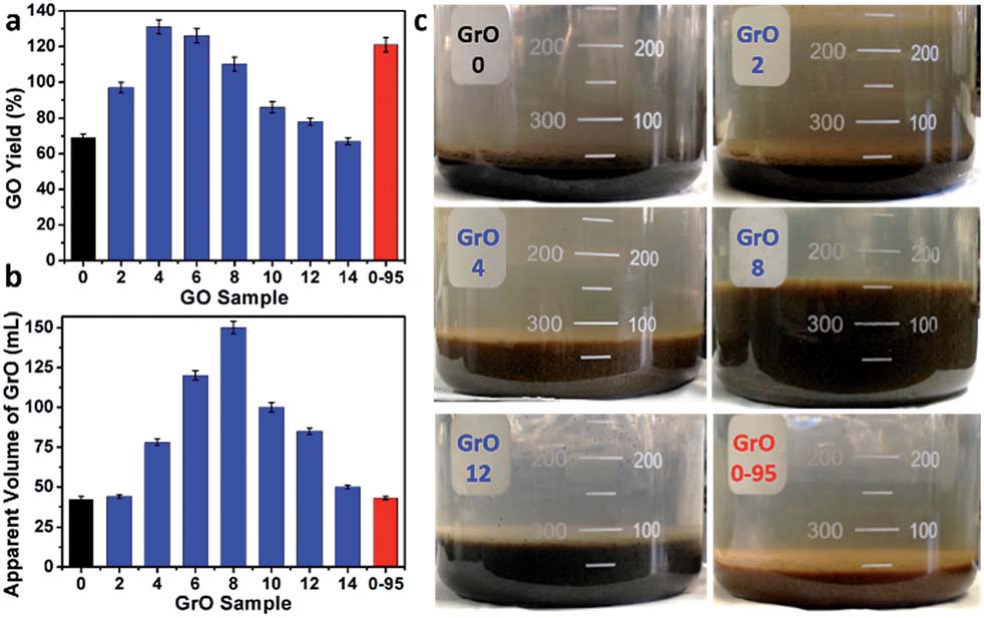
(a) Yields of GO-*n* and GO-0-95, and (b) the apparent volumes of their GrO precursors, (c) photographs of GrO-*n* and GrO-0-95 pastes; the values of *n* are depicted in the axis of (a) or (b), and in panel (c).

The yields of different GO samples have a large difference. The yield increased from 69 ± 2% for GO-0 to 131 ± 4% for GO-4, followed by a gradual decrease to 67 ± 2% for GO-14 (Fig. 6a). This trend can be explained by the following two opposite effects of water addition: (i) the increase of GO yield by increasing its oxidation degree (detailed mechanism will be discussed later); (ii) the decrease of GO yield caused by the dilution of H_2_SO_4_ with water, lowering the intercalation efficiency of H_2_SO_4_/KMnO_4_ between the graphene layers of graphite.^34^ Specifically, the enhanced oxidation played the dominant role as the water volume ≤ 4 mL, while dilution-induced insufficient intercalation became the crucial effect upon further addition of water. Actually, stage I graphite intercalation compound (GIC) of H_2_SO_4_ has alternate graphene and H_2_SO_4_ layers; it is an intermediate of converting graphite to GO.^35^ Stage I GIC of H_2_SO_4_ is usually formed by electrochemically or chemically increasing the electrochemical potential of graphite. The potential of stage I GIC (*E*_GIC-I_, indicative of the energy required for intercalation) increases, while the oxidizing potential of KMnO_4_ (*E*_KMnO_4__, indicative of the oxidation ability) decreases upon reducing the concentration of H_2_SO_4_ (*C*_H_2_SO_4__). Hence, stage I GIC could not be formed by oxidation with KMnO_4_ as *C*_H_2_SO_4__ < 14.4 M (close to the system with 14 mL water, *C*_H_2_SO_4__ = 14.8 M), because *E*_GIC-I_ < *E*_KMnO_4__.^34^ As a result, the yields of GO-10 to GO-14 were much lower than that of GO-4. Moreover, the GO-14 sample was found to have multilayered GrO sheets (Fig. S10†), possibly due to the insufficient intercalation discussed above.

On the other hand, the relatively high yield of GO-0-95 is attributed to the in-plane vacancies and holes formed during the destructive oxidizing of GrO at 95 °C, facilitating the osmotic swelling and exfoliation of GrO to individual GO sheets.

The ‘water-enhanced oxidation’ of graphite to GO in our systems is an unusual phenomenon, because water is an ineffective oxidant for graphite. Moreover, it was reported that ‘pristine GO’ reacted with water to form conventional GO with an increase of sp^2^-carbon content,^24^ contradictory to our observation of the water-induced higher oxidation degree of GO. These facts conclude that the ‘water-enhanced oxidation’ was not caused by the reaction of water with graphite or ‘pristine GO’.

In fact, GO samples were formed by the exfoliation of their GrO precursors (Method in ESI†). We found that the stable apparent volumes of the GrO pastes (kept undisturbed overnight for stabilization) formed by the oxidation of graphite in different systems (GrO-*n* and GrO-0-95) showed large variations (Fig. 6b and c). The volumes of GrO-*n* (*n* = 4 to 14) were measured to be 50–150 mL, much larger than those of GrO-0, GrO-2, and GrO-0-95 (42–44 mL). These volumes were also much larger than that of graphite powder (∼1.9 mL, Fig. S11†). The large volume expansion from graphite to GrO pastes is indicative of the partial exfoliation of GrO.^36^ The exfoliation of GrO is usually performed by enlarging the spaces between adjacent GO layers *via* releasing gases, forming ice crystals, or osmotic-swelling induced intercalation of water or other solvents. Experimentally, this phenomenon had already occurred during the 40 °C oxidation of graphite in concentrated H_2_SO_4_ as indicated by the extremely high viscosity of the GrO-8 paste (Fig. S12†).^37^ This exfoliation should be caused by gas-formation between adjacent graphene sheets of graphite or GrO, because osmotic swelling of GrO usually occurs in aqueous media with much higher pH values.^36^ The origin of the gas is discussed as follows. In the systems of forming GrO-0 and GrO-0-95, O_2_ was the predominant gas generated by the thermal decomposition of Mn_2_O_7_. However, in the systems of producing GrO-*n* (*n* = 2–14), O_3_ was generated *via* oxidizing water by multi-nuclear Mn(vii) clusters as reported in literature.^38^ The formation of O_3_ gas can also account for the larger volume expansions of GrO-*n* (*n* = 4–14) than those of the other GrO pastes.

**Fig. 7:**
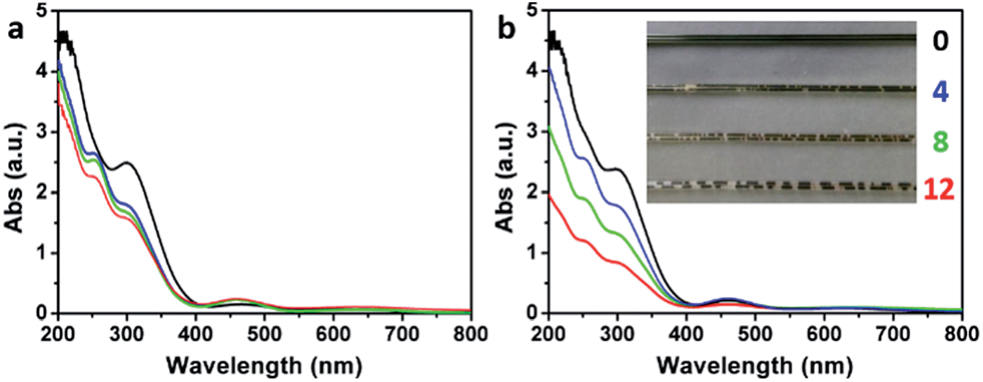
UV-Vis spectra of (a) fresh H_2_SO_4_ solutions of KMnO_4_ containing 0 (black), 4 (blue), 8 (green), and 12 mL (red) water at 0 °C, respectively, and (b) after keeping them at 40 °C for 2 h. Inset in (b) shows the photographs of the corresponding solutions in capillary tubes.

The formation of O_3_ by the addition of water into the concentrated H_2_SO_4_ solution of KMnO_4_ (the medium used for oxidizing graphite to GrO and GO) was also evidenced by monitoring the corresponding ultraviolet-visible (UV-Vis) spectra. The UV-Vis spectrum of a fresh concentrated H_2_SO_4_ solution of KMnO_4_ (3 g KMnO_4_ in 46 mL H_2_SO_4_) did not show a O_3_ band centered at 255 nm (Hartley band, Fig. 7a), while it showed a weak O_3_ band after being kept at 40 °C for 2 h (Fig. 7b), possibly resulting from the trace amount of water in concentrated H_2_SO_4_. In comparison, the spectra of the solutions containing 4, 8, or 12 mL of water showed a strong Hartley band both at its fresh state and after the heating treatment. Simultaneously, the absorptions of Mn(vii) with peaks at 300 and 460 nm^39^ decreased with the increasing content of water. The decrease in absorptions at 300 and 460 nm after the heating treatment were mainly attributed to the reduction of Mn(vii) by water, although the dilution of the solutions by adding water also had some contributions (only a maximum decrease of 16.3% by diluting with 12 mL water). The volume of the O_3_ gas formed in the solution also increased with its content of water, indicated by the numbers of gas bubbles generated in the solution filled in a capillary tube (inset of Fig. 7b). The solution without water addition is uniform with a deep green color of Mn(vii) (mainly from MnO_3_^+^). However, the solutions with water addition contain brown particles of MnO_2_ formed by reducing Mn(vii) with water.^40^

O_3_ is a well-known strong oxidant with a standard oxidizing potential (2.08 V) higher than that of MnO_4_^–^ (1.68 V).^41^ Moreover, the MnO_2_ formed by reducing Mn(vii) with water is an effective catalyst for decomposing O_3_ to atomic oxygen.^42^,^43^

**Fig. 8:**
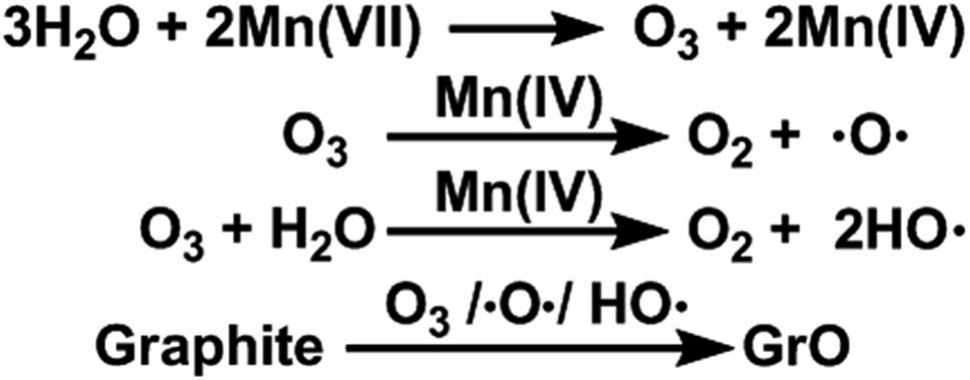
Proposed mechanism for water-enhanced oxidation of graphite.

Furthermore, the oxidation ability of molecular O_3_ can be enhanced by both water and newly-formed MnO_2_ (ref. 40) *via* the generation of hydroxyl radicals (HO˙).^44^,^45^ Actually, O_3_ (ref. 46), atomic oxygen,^47^ and HO˙ radicals^48^ are capable of oxidizing graphite to GrO, the precursor of GO. Especially, epoxy and hydroxyl groups can be formed by directly attacking graphene sheets with atomic oxygen and HO˙ radicals. On the basis of the above observations and discussion, the ‘water-enhanced oxidation’ of graphite to GrO can be attributed to the water-induced formation of strong oxidative species in the presence of Mn(vii) and H_2_SO_4_ (Fig. 8). The oxygenated groups of GrO were inherited to the corresponding GO.

On the other hand, the mechanism for the destructive oxidation process at 95 °C is elucidated as follows. Experimentally, 2.2–2.4 mL H_2_O_2_ (30%) was added to convert the remaining Mn species completely to soluble Mn(ii) ions in the system of preparing GO-0. However, in the system of preparing GO-0-95, only about 0.5 mL of H_2_O_2_ was consumed. Consequently, GrO-0-95 was further destructively oxidized by the residual Mn(vii) compound at an elevated temperature of 95 °C in a diluted H_2_SO_4_ solution (about 6.0 M). This destructive oxidation led to the formation of more permanent defects capped by carboxyl groups.

The ‘water-enhanced oxidation’ was observed to be more pronounced by lowering the temperature of the oxidizing graphite (ESI†). For example, the yield of the GO sample synthesized from the system with 4 mL water by oxidizing graphite at 0 °C for 48 h (GO-4-0-48 h) was measured to be about 60 times higher than that prepared from the system without adding water under the same conditions. Nevertheless, the quality of GO-4-0-48 h is much higher than those of GO-*n*. The electrical conductivity of rGO-4-0-48 h (894 ± 26 S cm^–1^) is much higher than that of rGO-0 (605 ± 20 S cm^–1^). Therefore, water addition is also an effective method to increase the oxidation degree of GO at low temperatures for producing high-quality GO in a much higher yield.

## Conclusions

The addition of a certain amount of water into the system of synthesizing GO *via* a modified Hummers method can increase the oxidation degree of GO sheets. This approach can also modulate the content of hydroxyl and epoxide groups on GO sheets without sacrificing their structural integrity, and greatly increase the yield of high-quality GO prepared at a low temperature of 0 °C. The selective formation of carboxyl groups on GO sheets has been realized by the destructive oxidizing of GO at a high temperature of 95 °C. This work provided a simple and scalable technique for producing GO with controlled species of functional groups.

## Supplementary Material

Supplementary information
