# Supplementary material for: Water-enhanced oxidation of graphite to graphene oxide with controlled species of oxygenated groups
Source: Chem Sci. 2015 Nov 26;7(3):1874–81. doi: 10.1039/c5sc03828f (PMC5965060; doi:10.1039/c5sc03828f)
Supplement: Supplementary file 1 [file SC-007-C5SC03828F-s001.pdf]

Electronic Supplementary Material (ESI) for Chemical Science.
This journal is © The Royal Society of Chemistry 2015

Electronic Supporting Information

Water-Enhanced Oxidation of Graphite to Graphene Oxide with Controlled Species of Oxygenated Groups

Ji Chen, Yao Zhang, Miao Zhang, Bowen Yao, Yingru Li, Liang Huang, Chun Li, and Gaoquan Shi*

Country Collaborative Innovation Center for Nanomaterial Science and Engineering, Department of Chemistry, Tsinghua University, Beijing 100084, People's Republic of China.

1. Methods

Preparation of GO at 40 °C. Natural graphite powder (100 mesh) with particle sizes in the range of 10 to 200 μm and 96.9 at% carbon (Figure S19) was purchased from Qingdao Huatai Lubricant Sealing S&T Co. Ltd. (Qingdao, China). Water (0, 4, 8 or 12 mL) was mixed with ice-cooled concentrated sulfuric acid (46 mL, < 10 °C) in a 250 mL flask setting in an ice bath with mechanical agitation at 200 r.p.m. After cooling down the solution to < 10 °C, graphite powder (1.0 g) was added and then $KMnO_4$ (3.0 g) was slowly added to keep the temperature of the suspension < 20 °C. Successively, the reaction system was transferred to a 40 °C oil bath and vigorously stirred (300 r.p.m.) for 2 h. In the cases of synthesizing GO-0 to GO-14, each of the oxidation product was slowly poured into 300 mL ice/water mixture under agitation to keep the temperature <10 °C, and the resulting solution was stirred for 15 min. For synthesizing GO-0-95, 100 mL water was slowly added to the reaction system after 40 °C oxidation in $H_2SO_4/KMnO_4$, followed by increasing the temperature of the solution to about 95 °C and maintained for 15 min. Subsequently, the reaction mixture was poured into 250 mL water. In all the cases, 5 mL H_2O_2 (30 %) was then added dropwise, turning the color of the solution from dark brown to yellow. Afterward, the suspension was kept undisturbed overnight to measure the apparent volume of GrO. The mixture was then filtered and washed with 1:9 HCl aqueous solutions (50 mL 3 times) to remove metal ions. The resulting solid was dried in air and dispersed in 500 mL water to form a GO aqueous dispersion. Finally, it was purified by dialysis for one week using a dialysis bag with a molecular weight cut off of 8,000 to 14,000 g

mol^{-1} to remove the remaining acid and metal species. The resultant GO aqueous dispersion was then stirred overnight to exfoliate it to GO. Successively, the GO dispersion was then centrifuged at 3000 r.p.m. for 20 min twice to remove the unexfoliated particles.

Preparation of GO at 0 °C. Water (0 or 4 mL) was mixed with ice-cooled concentrated sulfuric acid (46 mL, < 5 °C) in a 250 mL flask setting in an ice bath with mechanical agitation at 200 r.p.m. After cooling the mixture to < 5 °C, 1 g of natural graphite powder was added, and then 3 g $KMnO_4$ was slowly put into the system within 30 min. Successively, the reaction mixture was mechanically stirred (300 r.p.m) for 12 or 48 h under water bath cooling to about 0 °C by refrigerator system. After oxidation, the reaction mixture was poured into 300 mL ice/water mixture to keep the temperature < 10 °C. The resulting solution was stirred for 15 min, and followed by the addition of 5 mL H_2O_2. The washing and dialysis processes were the same to those described above. Afterward, the GO dispersion was then centrifuged at 3000 r.p.m. for 20 min 5 times to remove the unexfoliated particles and used for further comparisons. To obtain large-size GO sheets, centrifugation rate of 2000 r.p.m. should be applied to avoid precipitation of lager GO sheets at higher centrifugation rates. The SEM images in Figure S18 demonstrate the large-size GO sheets synthesized at 0 °C for 48 h with adding 4 mL water and finally centrifuged at 2000 r.p.m.

Preparation of GO and rGO Papers. The GO films were prepared by drying 5 mg mL^{-1} GO suspensions in plastic containers placed in a glass dryer at room

temperature. The thickness of each GO film was controlled to be about 6−7 μm by adjusting the volume of GO suspension and the size of container. rGO films were obtained by chemical reduction of GO films by immersing them in a 1:1 (by volume) mixture of HI solution (57 wt. %) and ethanol sealed in a container at room temperature overnight. The rGO films were then repeatedly washed with water and ethanol, and finally dried at room temperature overnight for characterizations.

Measurements of GO yields. A certain volume of purified GO aqueous dispersion was freeze-dried for over 48 h, and the weight of dried GO was used to calculated the concentration of GO (C_{GO} in mg mL^{-1}). The yield of GO (Y_{GO}) was calculated by $Y_{GO} = (C_{GO} \times V_{GO}/m_{Gr}) \times 100\%$, where V_{GO} is the total volume of purified GO dispersion (in mL), m_{Gr} is the weight of feeding graphite powder (in mg).

UV-vis spectral studies on the oxidative solutions. A certain volume of water (0, 4, 8, or 12 mL) was mixed with ice-cooled concentrated sulfuric acid (46 mL, < 10 ^{o}C) in a 250 mL flask setting in an ice bath with mechanical agitation at 200 r.p.m. After cooling down the solution to < 10 ^{o}C, 3 g of $KMnO_4$ was slowly added and stirred for 15 min under ice bath. Successively, the reaction system was transferred to a 40 ^{o}C oil bath and the stirring rate was increased to 300 r.p.m. Samples were taken out from the reaction system after heating for 0 or 2 h, and diluted for recording their UV-vis spectra.

Characterizations. X-ray photoelectron spectra (XPS) were carried out by the use of an ESCALAB 250 photoelectron spectrometer (ThermoFisher Scientific) with Al Kα (1486.6 eV) as the X-ray source set at 150 W and a pass energy of 30 eV for high

resolution scan. Solid-state ^{13}C magic-angle spinning (MAS) NMR experiments were conducted on a Bruker Avance-III spectrometer (100.6 MHz ^{13}C, 400.1 MHz ^{1}H) and a 4 mm MAS rotor probe. Direct ^{13}C pulse spectra were acquired using a MAS spinning speed of 10,000 Hz and ~10,000 scans. Raman spectra and optical images were recorded on a LabRAM HR Evolution (Horiba Jobin Yvon) with a 514-nm laser. Electrical conductivity was measured by a four-point probe technique. The rGO films were directly used for structural characterizations. UV-visible spectra were taken out by the use of a Lambda 35 UV-visible spectrometer (PerkinElmer, USA). Scanning electron micrographs (SEM) were performed by the use of a field-emission scanning electron microscope (Sirion-200, Japan). The samples used for SEM characterizations were prepared by depositing ethanol-diluted (5 μg mL^{-1}) GO aqueous solutions onto 300 nm SiO_2/Si wafers (washed by alternative sonication in acetone/ethanol for 3 cycles and stored in ethanol). The sheet sizes were measured to be the mean value of the length and width of an individual GO sheet. Attenuated total reflectance-Fourier transform infrared spectroscopy (ATR-FTIR) spectra were recorded on a Fourier transform infrared spectrometer (Bruker Vertex V70). X-ray diffraction (XRD) was carried out by the use of a D8 Advance X-ray diffractometer with Cu Kα radiation (λ=0.15418 nm, Bruker, Germany).

2. Supplementary Figures

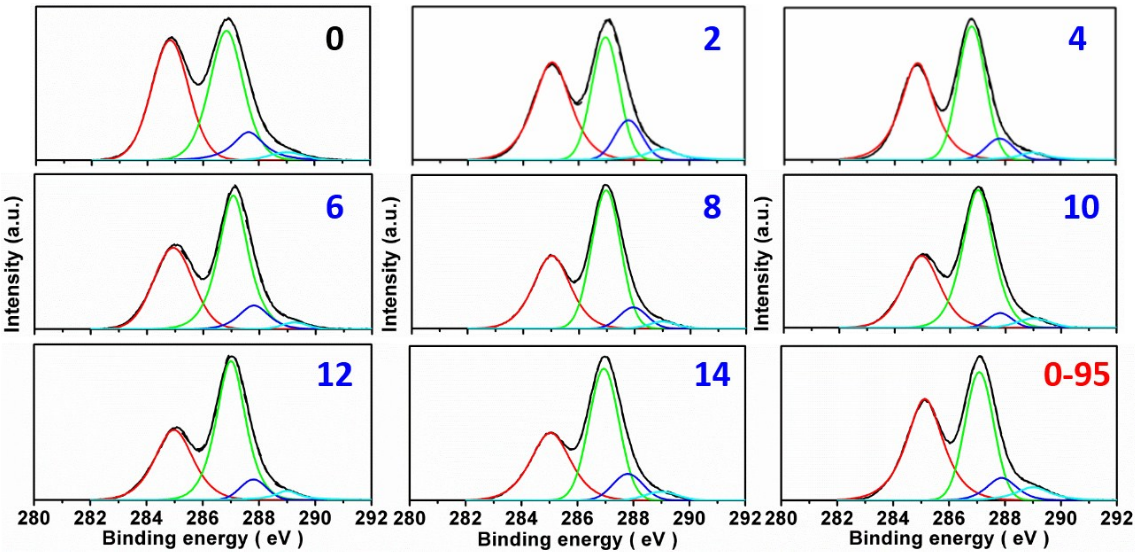

Figure S1. C 1s XPS spectra of freeze-dried GO-n and GO-0-95; the values of n are depicted in the figure.

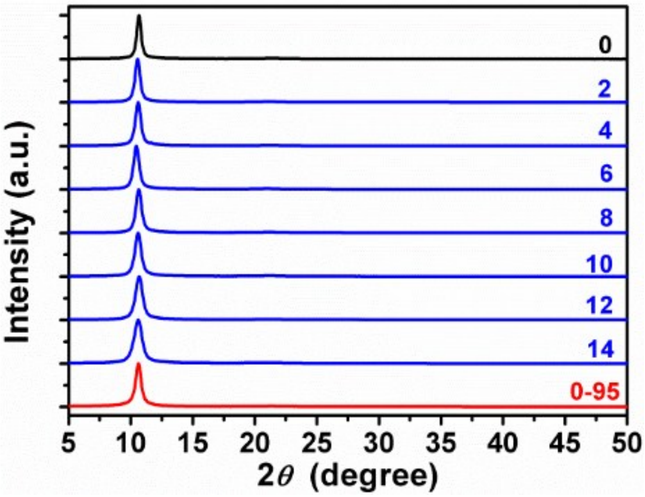

Figure S2. XRD patterns of dried films of GO-n and GO-0-95; the values of n are depicted in the figure.

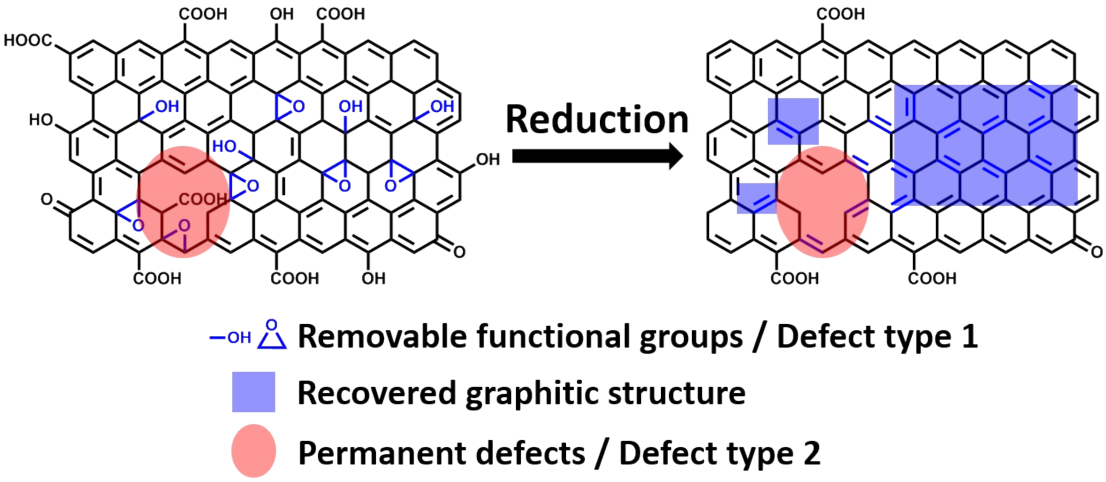

Figure S3. Schematic illustration of the two kinds of 'defects' in GO before and after reduction.

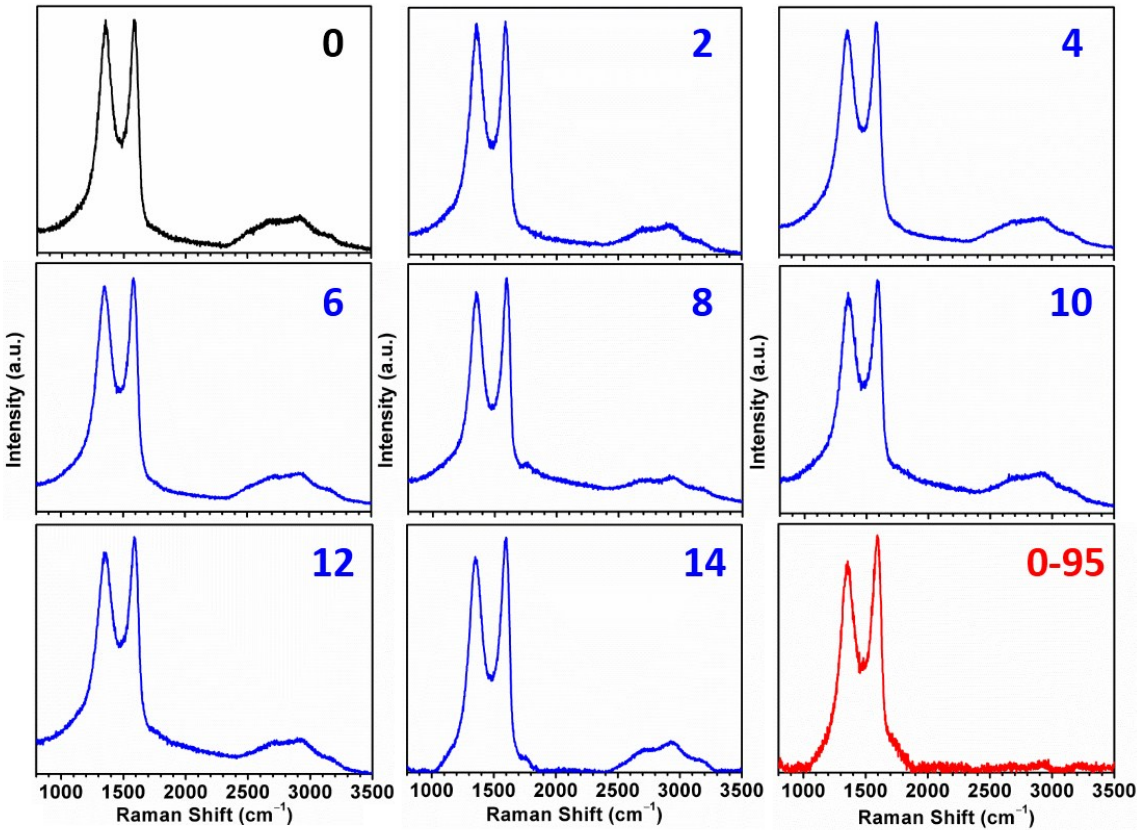

Figure S4. 514.5-nm excited Raman spectra of freeze-dried GO-n and GO-0-95; the values of n are depicted in the figure.

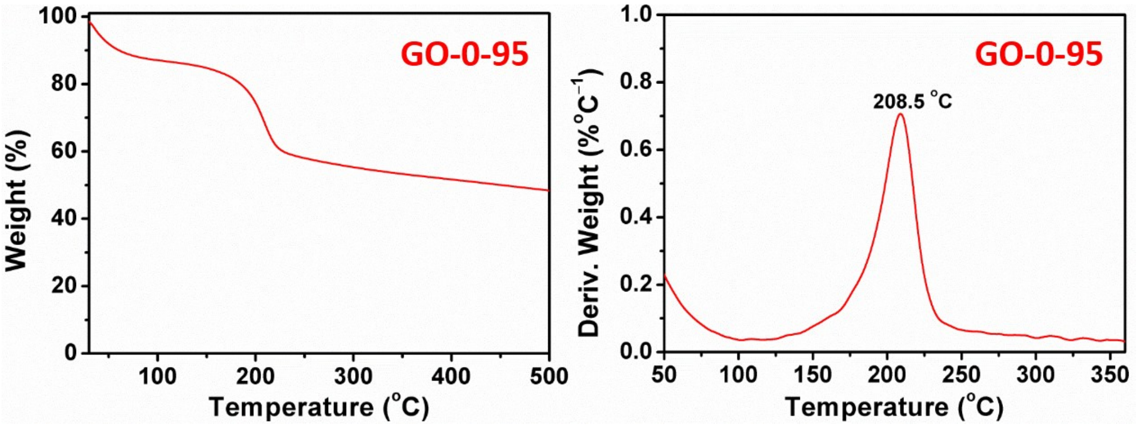

Figure S5. Thermogravimetric analysis curves of GO-0-95.

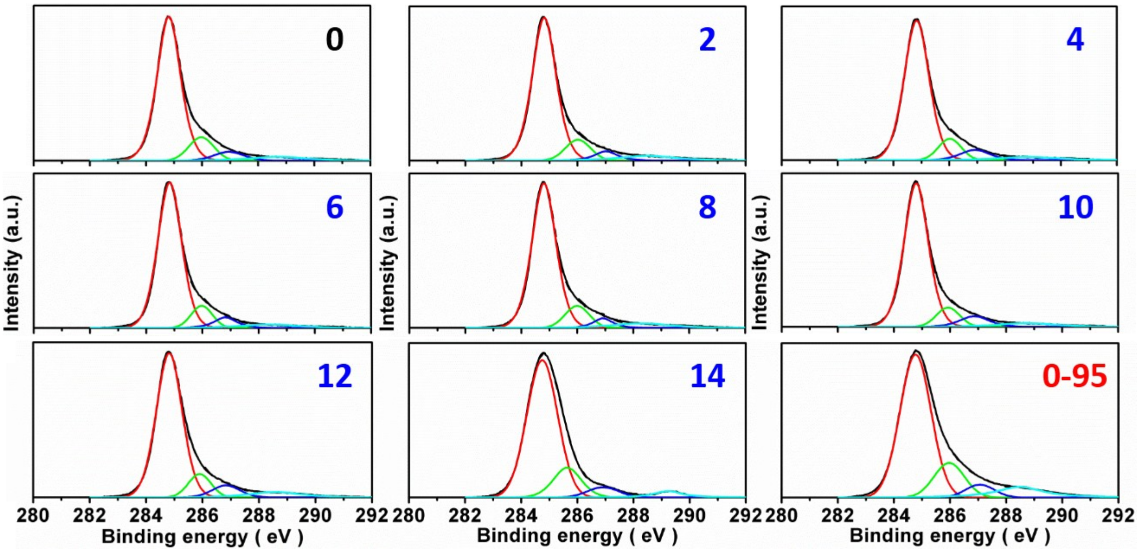

Figure S6. C 1s XPS spectra rGO-n and rGO-0-95; the values of n are depicted in the figure.

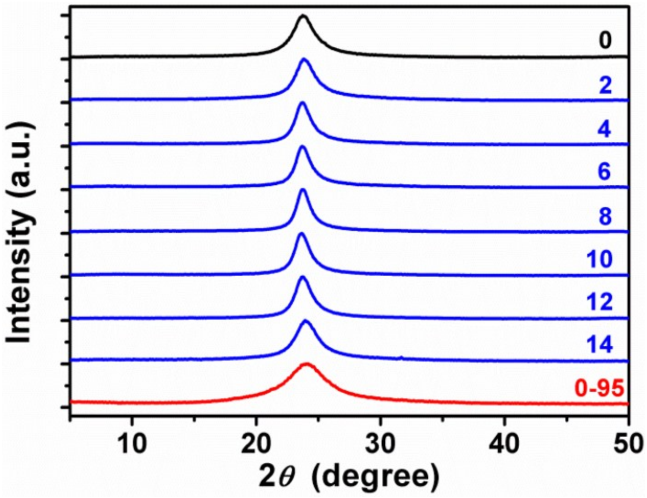

Figure S7. XRD patterns of rGO-n and rGO-0-95 papers; the values of n are depicted in the figure.

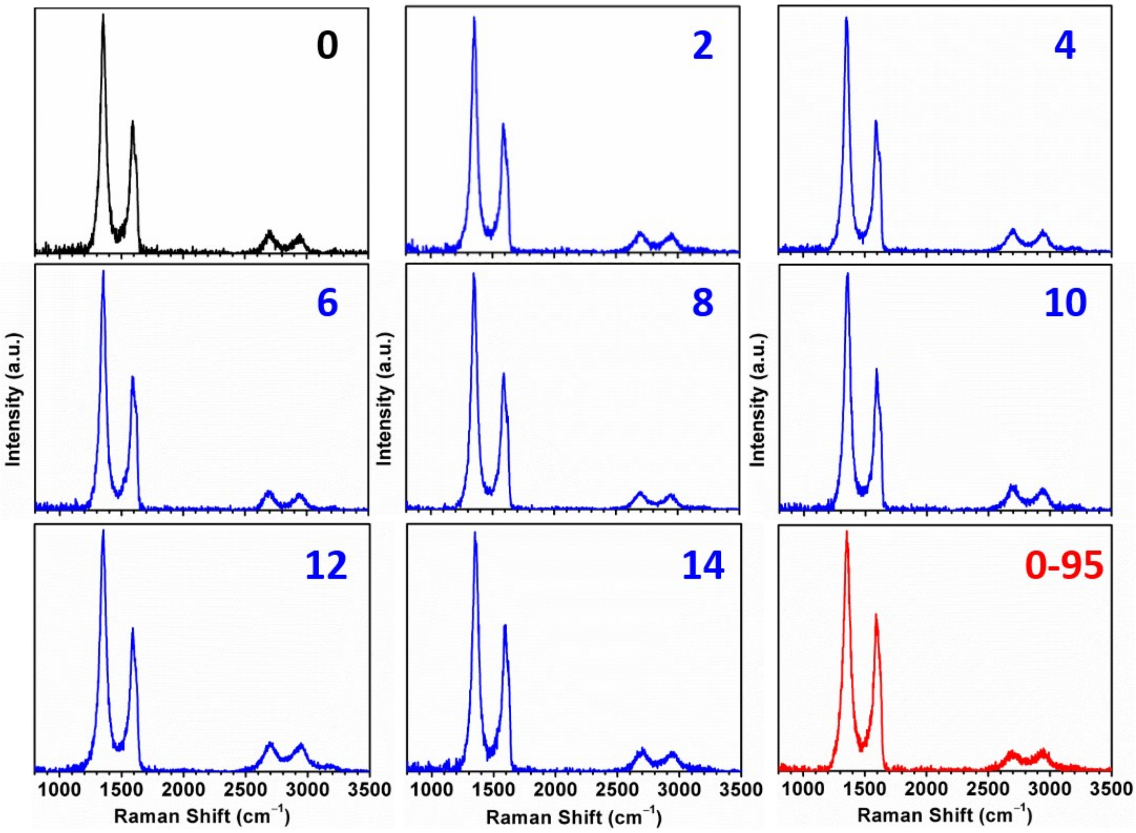

Figure S8. 514.5-nm excited Raman spectra of rGO-n and rGO-0-95 papers; the values of n are depicted in the figure.

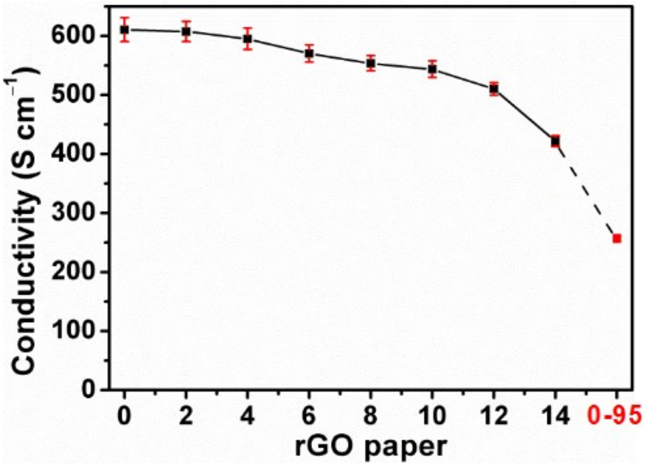

Figure S9. Conductivities of rGO-n and rGO-0-95 papers; the values of n are depicted in the X-axis.

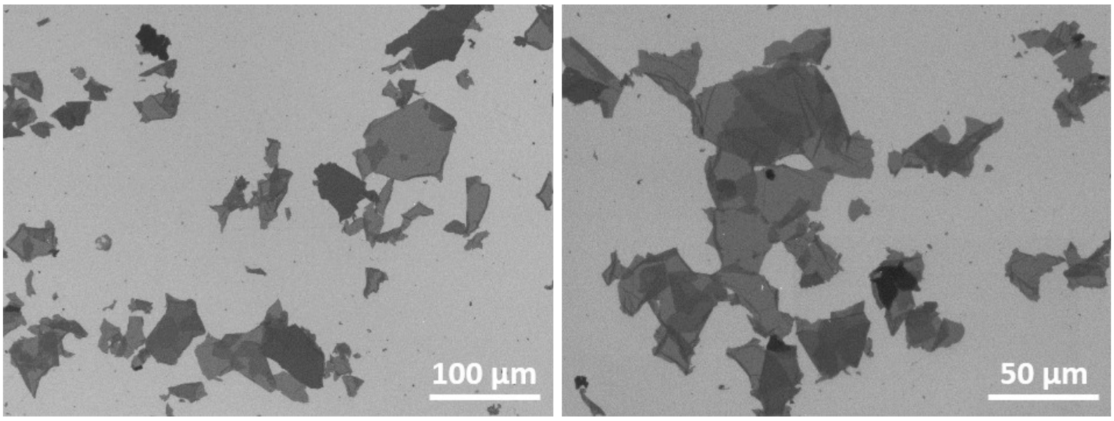

Figure S10. SEM images of GO-14.

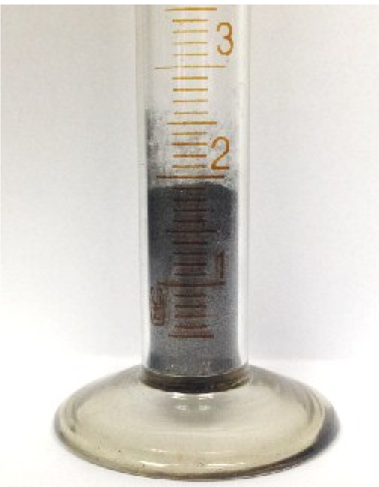

Figure S11. Photograph of 1 g 100 mesh natural graphite used for the preparation of GO.

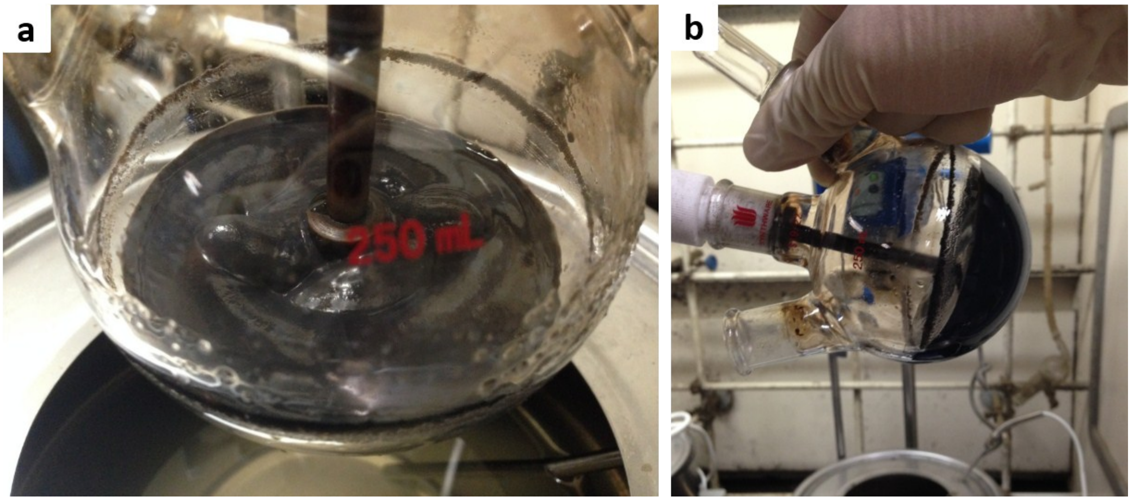

Figure S12. Photographs of the reaction system after oxidizing graphite to GrO-8.

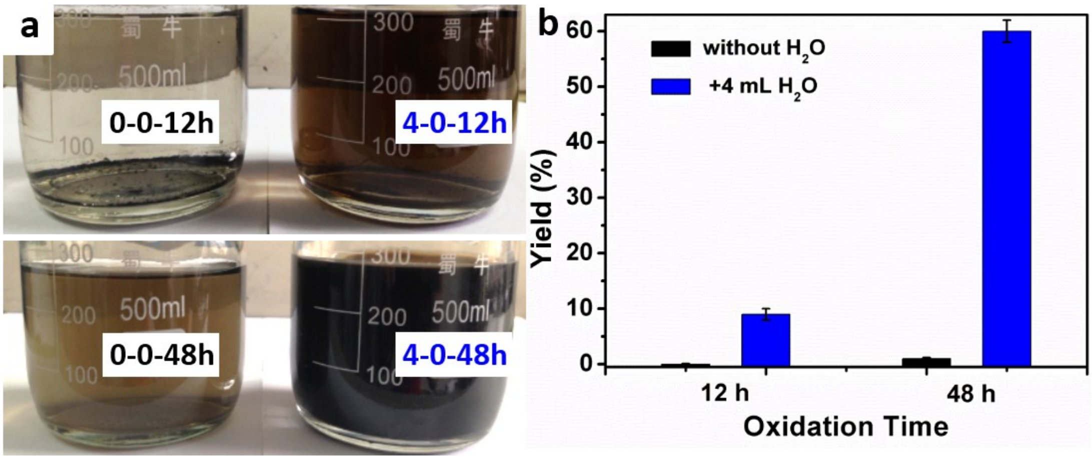

Figure S13. a) Photographs of the suspensions of GO-a-0-bh (a =0 or 4, and b= 12 or 48), and (b) the comparison of their yields.

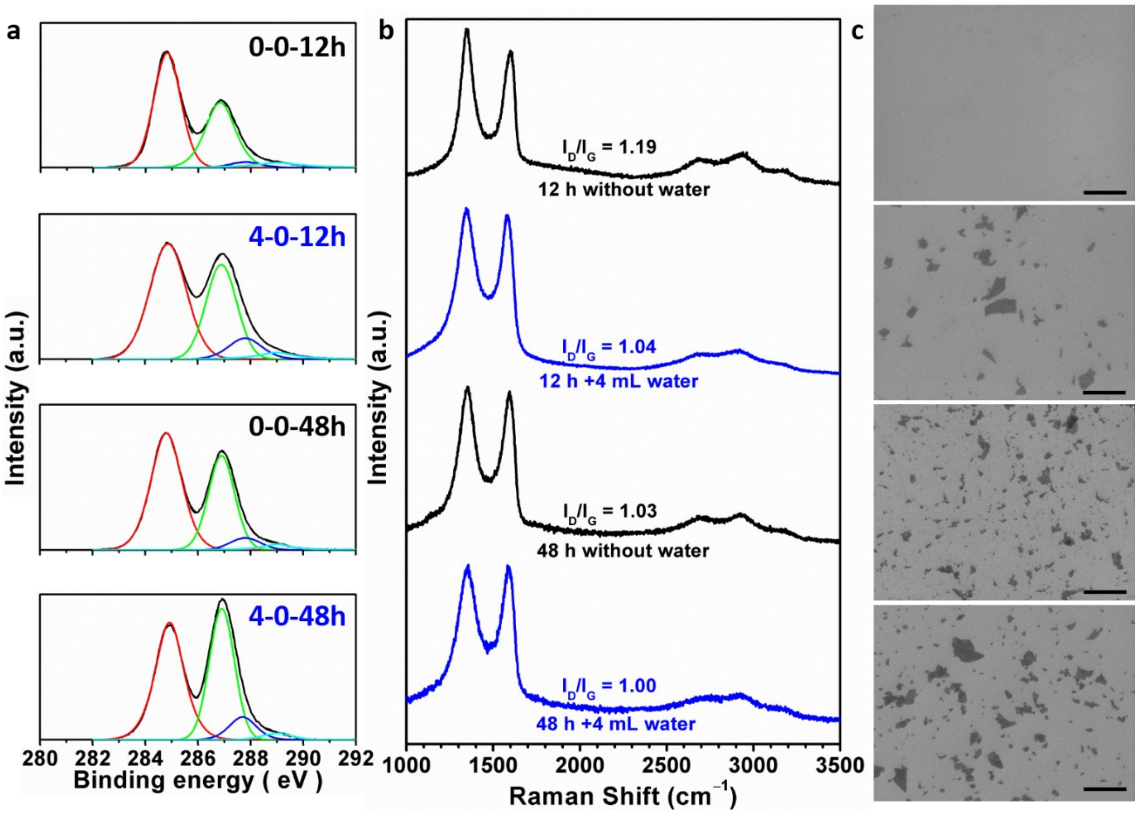

Figure S14. a) C 1s XPS spectra, b) 514.5-nm excited Raman spectra, c) typical SEM images of GO-a-0-bh samples (oxidation of graphite at 0 °C for b hours in the system containing a mL water); scale bar = 20 µm. Raman spectra and SEM images of the corresponding GO samples denoted in the most left panels.

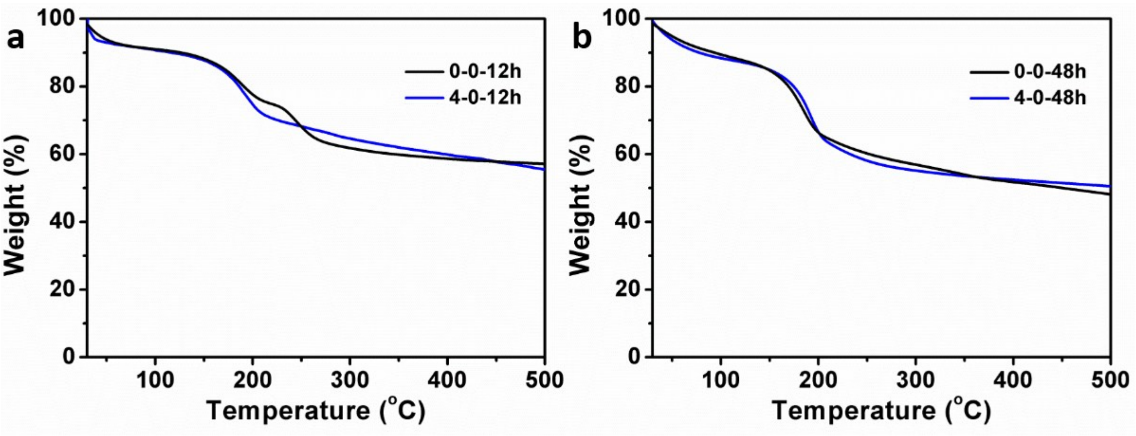

Figure S15. Thermogravimetric analysis curves of freeze-dried GO-a-0-bh samples.

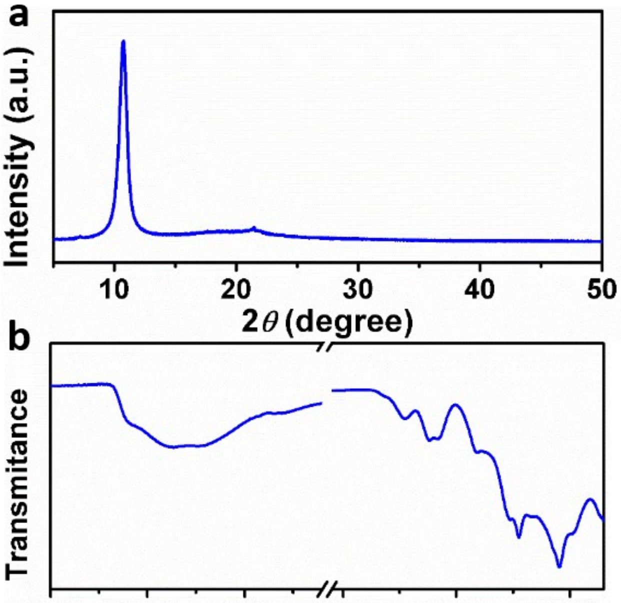

Figure S16. a) XRD pattern and b) ATR-FTIR spectrum of GO-4-0-48h samples.

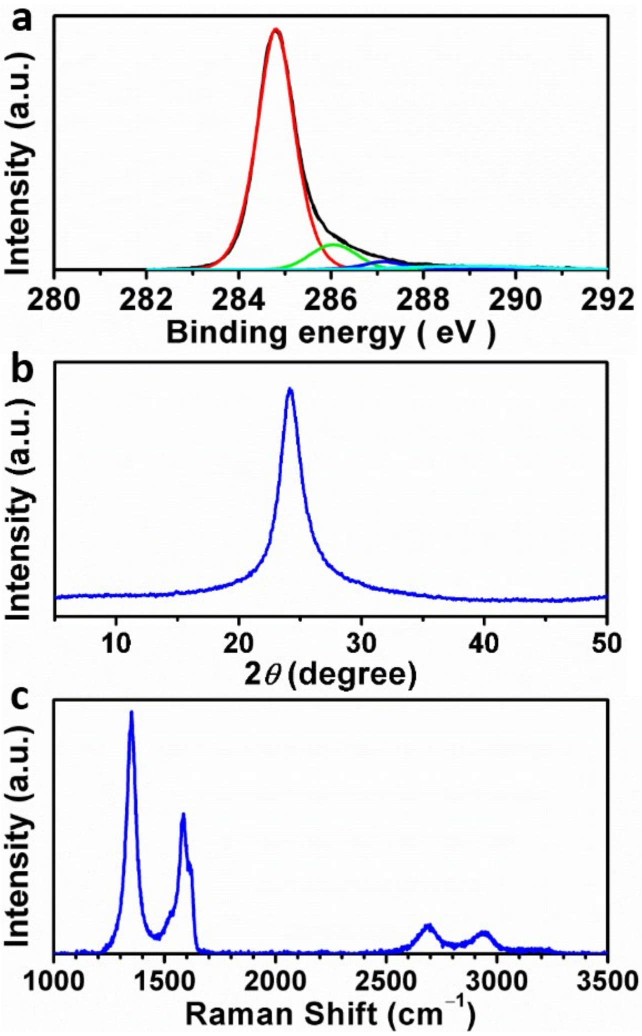

Figure S17. a) C 1s XPS spectrum, b) XRD pattern, and c) 514.5-nm excited Raman spectrum of rGO-4-0-48h paper prepared by HI reduction of GO papers made from modified Hummers methods with 4 mL water addition at 0 ^{o}C for 48 h.

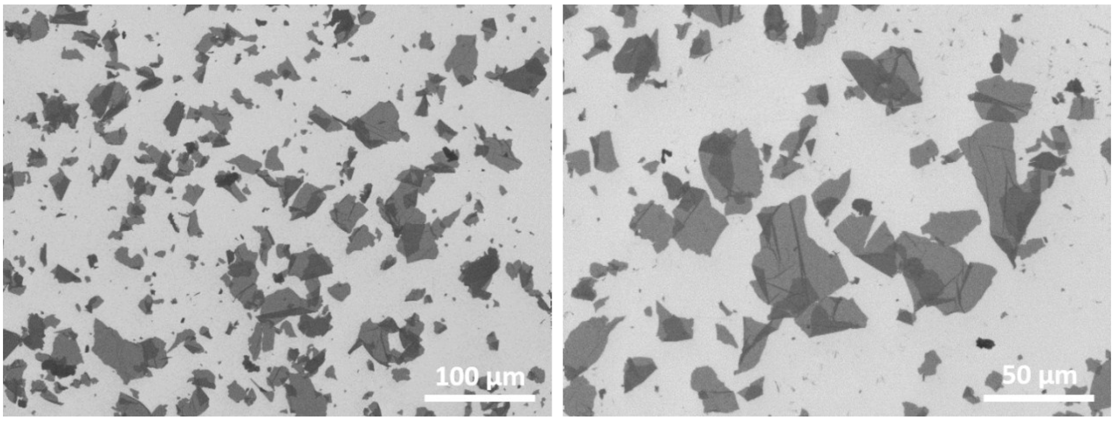

Figure S18. Typical SEM images of GO prepared at 0 °C for 48 h with adding 4 mL of water and centrifuged at 2000 r.p.m.

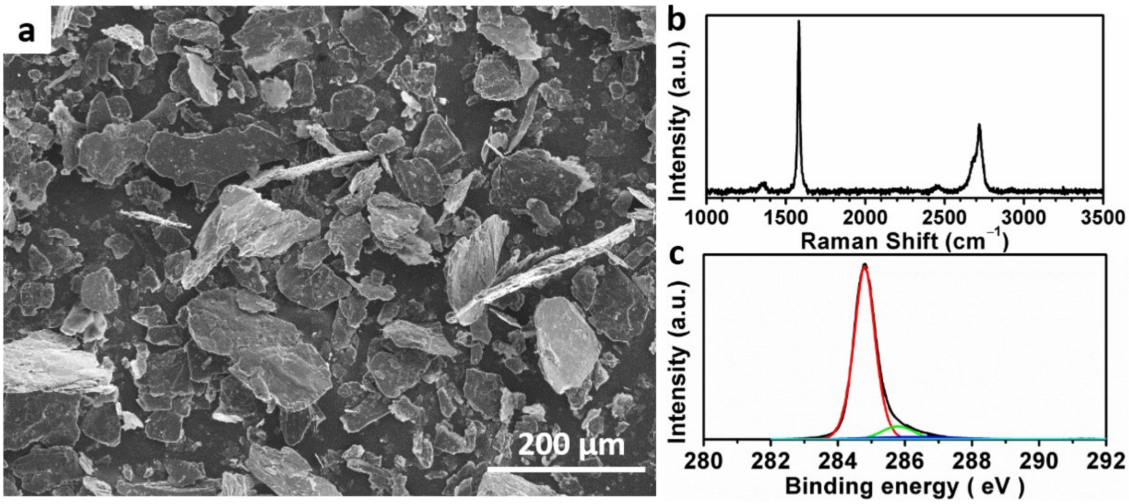

Figure S19. a) Typical SEM images, b) 514.5-nm excited Raman spectrum, c) C 1s XPS spectrum of the raw 100 mesh graphite powders.

3. Water-Enhanced Oxidation of Graphite to GO at 0 °C

Water-enhanced oxidation can greatly increase the yield of high-quality GO synthesized at 0 °C. For comparison, the GO samples were prepared by oxidation of graphite in the systems with addition of a mL (a = 0 or 4) water for b hours (b =12 or 48), and they were nominated as GO-a-0-bh. The yields of GO-4-0-bh were found to be much higher than those of the corresponding GO-0-0-bh, as reflected by the color difference of their dispersions (Figure S13a). Actually, the yields of GO-0-0-12h and GO-0-0-48h were measured to be only ~0 and 1 ± 0.3 %. However, the yields of GO-4-0-12h and GO-0-0-48h were dramatically increased to 9 ± 1 and 60 ± 2 % (Figure S13b). It should be noted here, the GO-0-0-12h product did not have monolayer GO sheets, composing of partially exfoliated particles. GO-0-0-12h completely precipitated from its dispersion upon keeping undisturbed for several days.

Water-induced increase of the yield of GO-a-0-bh was caused by increasing its oxidation degree. As shown in Figure S14a, the relative content of oxidized carbon atoms increased remarkably with the increase of water volume from 43.7 % for GO-0-0-12h to 47.9 % for GO-4-0-12h, and from 45.5 % for GO-0-0-48h to 53.7 % for GO-4-0-48h. Raman spectral studies indicated that the I_D/I_Gs of GO-a-0-bh samples decreased as ‘a’ changed from 0 to 4 (Figure S14b), also reflecting the increase of oxidation degree. SEM images (Figure S14c) showed that the sizes of GO-4-0-bh sheets are larger than those of GO-0-0-bh counterparts. The largest sheets of GO-4-0-bh sheets are larger than 10 μm, while those of GO-0-0-bh are smaller than 7 μm. This is mainly due to that the higher oxidation degrees GO-4-0-bh sheets facilitate the exfoliation from their GrO precursors.

The TGA curves of GO-a-0-bh are shown in Figure S15. Among them, the curve of GO-0-0-12h exhibits two steps of thermal decomposition in the temperature range of 150 to 300 °C, indicating the presence of organosulfate groups. This incomplete hydrolysis of organosulfate was resulted from unexfoliated GrO, because the

organosulfate groups in GrO matrix were inaccessible to water. The decomposition of organosulfate are not observed from the other TGA curves, indicating the corresponding GO samples have been well exfoliated and hydrolyzed. These TGA curves also indicate that the contents of functional groups in the GO samples are in the sequence of GO-0-0-12h < GO-4-0-12h ≈ GO-0-0-48h < GO-4-0-48h. XPS and Raman analysis also confirmed this observation.

The amounts of GO-0-0-12h, GO-4-0-12h, and GO-0-0-48h were insufficient for fabricating robust films because of their extremely low yields. Thus, only GO-4-0-48h paper was prepared and further reduced to conductive rGO-4-0-48h. The XRD pattern of GO-4-0-48h paper (Figure S16a) featured one characteristic GO peak at about 2θ= 10.76^o (d-spacing=8.23 Å), demonstrating the successful functionalization of graphene sheets. The ATR-FTIR spectrum of GO-4-0-48h (Figure S16b) shows similar feature compared with those GO synthesized at 40 ^{o}C except for the relatively weak peak for C=O (1740−1720 cm^{-1}) and a new peak for graphitic domains (~1580 cm^{-1}),[S1] indicating the better structural integrity. The XPS C 1s spectrum of rGO-4-0-48h indicates the successful removal of oxygenated functional groups (Figure S17a). The XRD pattern (Figure S17b) of rGO-4-0-48h exhibits single characteristic (002) reflection peak at 2θ = 24.2 ^{o}, corresponding to a d-space of 3.68 Å. This d-space is smaller than those of rGO-n (3.71−3.74 Å) because of its lower content of the residual oxygenated groups.

Typical Raman spectrum of rGO-4-0-48h (Figure S17c) features a stronger D-bands with respect to G-band (I_D/I_G = 1.72), indicating the restoration of graphitic structures.[S2, S3] Furthermore, the G band of rGO-4-0-48h is a combination of a narrow G peak centered at ~1580 cm^{-1} and a broader blue-shifted peak (G+D'), having a higher intensity ratio of $I_G/I_{D'}$ with respect to that of rGO-0.[S4] Moreover, the intensity ratio (I_{2D}/I_{D+G}) of 2D band (~2690 cm^{-1}) to D+G band (~2940 cm^{-1}) of rGO-4-0-48h was measured to be 1.31, much higher than that (1.03) of rGO-0.[S4] This result implies that the structural integrity of rGO-4-0-48h sheets is better than that of rGO-0 sheets.

It is also worth noting that the GO synthesized at 0 °C is less dispersible than those synthesized at 40 °C because of its better structural integrity and less hydrophilicity. Therefore, a low speed of centrifugation (~2000 r.p.m.) should be adopted to obtain large-sized GO sheets. The GO-4-0-48h sheets collected from their dispersion by centrifugation at 2000 r.p.m. (Figure S18) showed an average size of ~ 14.5 μm, larger than that of the GO sheets collected by centrifugation at 3000 r.p.m. (Figure S14c).

4. Supplementary References

(S1) P. V. Kumar, N. M. Bardhan, S. Tongay, J. Wu, A. M. Belcher and J. C. Grossman, *Nat. Chem.*, 2014, **6**, 151-158.

(S2) S. Eigler, C. Dotzer, F. Hof, W. Bauer and A. Hirsch, *Chem. Eur. J.*, 2013, **19**, 9490-9496.

(S3) S. Eigler, C. Dotzer and A. Hirsch, *Carbon*, 2012, **50**, 3666-3673.

(S4) H. Wang, J. T. Robinson, X. Li and H. Dai, *J. Am. Chem. Soc.*, 2009, **131**, 9910-9911.
